# Regenerative Radio Electric Asymmetric Conveyer Treatment in Generalized Cerebral and Cerebellar Atrophy to Improve Motor Control: A Case Report

**DOI:** 10.7759/cureus.28245

**Published:** 2022-08-21

**Authors:** Salvatore Rinaldi, Chiara Rinaldi, Vania Fontani

**Affiliations:** 1 Department of Research, Rinaldi Fontani Foundation, Florence, ITA; 2 Department of Regenerative Medicine, Rinaldi Fontani Institute, Florence, ITA; 3 Department of Neuroscience, Psychology, Drug Area and Child Health (NEUROFARBA), University of Florence, Florence, ITA

**Keywords:** reac, neurobiological stimulation, neuromodulation, neurostimulation, endogenous bioelectrical fields, endogenous bioelectrical activity, cerebellar atrophy, cerebral atrophy

## Abstract

This report presents a case with a diagnosis rarely described in the literature, that is generalized cerebral-cerebellar atrophy. The patient showed a rapid decline with general cognitive deterioration, memory loss, temporal and spatial disorientation, and ataxic manifestations in voluntary movements. The loss of neurons and synaptic connections can be explained by an alteration of the correct endogenous bioelectrical activity (EBA), the phenomenon which allows all the processes of cellular life, such as differentiation, proliferation, migration, morphogenesis, apoptosis, and neurotransmission. The patient was treated with a specific regenerative neurobiological stimulation treatment applied with the radio electric asymmetric conveyer (REAC) technology, which was designed to recover the correct EBA. The tissue optimization regenerative (TO RGN) treatments used in this case report have already demonstrated the ability to induce neuroregenerative processes. At the follow-up, the patient showed a reduction in ataxia both in walking and running. This case report allows us to learn that the manipulation of the EBA can induce improvements even in clinical cases in which the scientific literature leaves no room for improvement.

## Introduction

Cerebral atrophy is a progressive and permanent reduction of brain tissue, and it can be focal when the damage affects a specific area of the brain [1]. Sometimes, atrophy can involve one or more brain structures, such as the cerebellum. In this case, we have generalized cerebral-cerebellar atrophy, rarely described in the literature, so much so that it does not have its own International Classification of Diseases 10th Revision (ICD-10) code. Cerebral atrophy is characterized by both the loss of neurons and their synaptic connections (SCs). The SCs constitute the bioelectrochemical connectors between neurons, consequently, the progressive loss of the SCs determines the decay of neurotransmission and therefore decay of neuronal functionality and vitality. The basis of neurotransmission is endogenous bioelectrical activity (EBA). In addition to allowing all the processes of cellular life, such as differentiation, proliferation, migration, morphogenesis, and apoptosis, the EBA also allows the processes of neurotransmission [2-6]. The loss of the correct EBA could therefore be the initial cause that would determine the loss of synapses and neurons [7]. To recover EBA, a neurobiological stimulation technology called radio electric asymmetric conveyer (REAC) was studied, which is administered through specific treatment protocols in relation to the pathology to be treated [8,9]. In the case presented here, the tissue optimization regenerative (TO RGN) treatments were used, which demonstrated the ability to induce neuroregenerative processes [10-13], even in the case of chemical destruction of specific neurons such as those of the substantia nigra [14]. The REAC medical device used in this study was the BENE 110 (Florence, Italy: ASMED SRL).

## Case presentation

The patient was a 79-year-old university-educated man who has always held senior management roles throughout his career. He was a long-time sufferer of type II diabetes and hypertension, in pharmacological treatment with oral hypoglycemic and antihypertensive drugs. The patient had not performed genetic testing, and the wife denied familiarity. Since the patient came to us with a complete medical record, completed the previous week, we did not consider it appropriate to repeat psychometric tests or reassess the ataxia with specific scales.

Over the past three years, the patient has shown rapid decline with general cognitive deterioration, memory loss, and temporal and spatial disorientation. In the last year, the decline has increasingly involved the neuromotor component with ataxic manifestations. A magnetic resonance examination showed significant cerebral and cerebellar atrophy, drawing a picture of generalized cerebral-cerebellar atrophy. On the recommendation of medical relatives, the patient was sent to our institute, where we were able to ascertain the evident overall neurocognitive decay and the neuromotor ataxic component. Ataxia was evident in various voluntary movements, such as carrying food to the mouth but also in walking (Video 1) and even more in attempting to run (Video 2).

**Video 1:** Patient walking before REAC treatments. REAC: radio electric asymmetric conveyer

**Video 2:** Patient attempting to run before REAC treatments. REAC: radio electric asymmetric conveyer

During the first visit, the patient underwent a preliminary REAC neurobiological modulation treatment called Neuro Postural Optimization (NPO), aimed at inducing an initial brain remodulation and treating functional dysmetria [15-17]. The REAC NPO is a preprogrammed single-session treatment of a few milliseconds. It is administered by applying the tip of the metallic REAC asymmetric conveyer probe (ACP) to a specific area of the ear pavilion.

Subsequently, the patient underwent REAC TO RGN type N treatment for a total of 25 hours, administered five hours a day for five consecutive days [14]. The REAC TO RGN type N is a preprogrammed treatment, that the operator cannot modify in any parameter. The treatment is administered by placing an ACP along the spine holding it in place with a tubular elastic net. At the follow-up performed after about 30 days, the patient showed an initial improvement in interpersonal skills, but above all a clear reduction in ataxia both in walking (Video 3) and running (Video 4).

**Video 3:** Patient walking after REAC treatments. REAC: radio electric asymmetric conveyer

**Video 4:** Patient running after REAC treatments. REAC: radio electric asymmetric conveyer

## Discussion

Neurodegenerative diseases, linked to senescence, are a constantly growing phenomenon linked above all to the increase in life expectancy. Unfortunately, there are no effective pharmacological treatments for these pathologies. For this reason, research in the various sectors of regenerative medicine is trying to find effective and safe solutions. In addition to genetic engineering, other methodologies are opening up new therapeutic perspectives for neurodegenerative diseases. Among these, the electroceutical [18] with various types of approaches and technologies seems to demonstrate a potential efficacy in countering neurodegenerative decay [7]. REAC technology represents one of these electroceutical technologies.

The ability to manipulate EBA in order to promote reparative and regenerative effects has long been considered essential [7]. Thanks to the continuous progress of technological research, it has only recently been possible to face this challenge positively. REAC technology has been designed for this purpose, demonstrating to be able to directly reprogram cell fate also towards neuronal differentiation [10-13], and at the same time fighting inflammatory phenomena that can aggravate neurodegenerative processes [14,19]. These results are clinically evident in the improvements observed in this case report.

Of course, these results, although positive, are the result of only one very short cycle of REAC RGN type N treatment. In fact, the expected duration of the treatment is at least 200 hours. This duration was inferred from previous animal model studies, in which neuronal death of specific parts of the brain was chemically produced [14]. Naturally, given the variability of the causes that can induce neurodegenerative phenomena such as generalized cerebral-cerebellar atrophy, further studies will be needed to establish the treatment duration protocols.

The fact that the REAC TO RGN treatments are non-invasive, painless, and easy to administer represents certainly an element of strength. The weaknesses of this study are those that all the case reports have in common. Unfortunately, due to family problems, the patient was unable to continue the treatments and consequently, our follow-up stops at 30 days. Although this period is very short, this initial significant improvement encourages us to continue to spread this new therapeutic possibility to patients who otherwise would have no other therapeutic perspectives.

## Conclusions

Regenerative medicine is an interesting field of medicine and is rapidly evolving. Since some genetic engineering and stem cell implantation techniques have shown little or no efficacy compared to expectations or side effects, research is looking for more effective and safer techniques. REAC RGN treatments have been designed to achieve these goals in regenerative medicine. Of course, further studies will be needed.
